# The course and outcome of renal failure due to human leptospirosis referred to a hospital in North of Iran; A follow-up study 

**Published:** 2016

**Authors:** Roya Ghasemian, Mehran Shokri, Atieh Makhlough, Mohammad Amin Suraki-Azad

**Affiliations:** 1 Antimicrobial Resistance Research Center, Mazandaran University of Medical Sciences, Sari, Iran.; 2 Infectious Diseases and Tropical Medicine Research Center, Babol University of Medical Science, Babol, Iran.; 3 Mazandaran University of Medical Science, Sari, Iran.

**Keywords:** Leptospirosis, Renal failure, Death, Renal outcome

## Abstract

**Background:**

Renal complication of leptospirosis is common and its clinical manifestations vary from urinary sediment changes to acute renal failure. The aim of this study was to determine the final outcome of renal involvement in leptospirosis.

**Methods:**

This longitudinal prospective study included all serologically confirmed cases of leptospirosis with evidence of renal failure. All patients were followed for three months while all patients with renal failure were followed-up for one year.

**Results:**

Fifty-one patients, 53.5±14.8 years (82.4% males) with acute renal failure were studied. Over the hospitalization period, 28 patients recovered, and seven (13.72%) patients died of multiple organ failure. At the time of discharge, 16 patients had mild renal failure. Over the follow-up period, all patients recovered but in two patients renal failure persisted at creatinine level of 1.5 mg/dl.

**Conclusion:**

Development of renal failure in leptospirosis is not rare. Recovery of renal function may last several months. However, most patients recover completely at least after one year.

Leptospirosis is probably the commonest infection worldwide presenting with a wide range of manifestations from asymptomatic disease to severe and lethal form renal involvement is an important feature of leptospirosis especially in severe forms (1). Most cases of leptospirosis are self-limited with excellent prognosis. However, up to 10% of leptospirosis infections may induce acute renal failure (ARF) and are associated with significant morbidity and mortality (1). Specifically in northern part of Iran due to large rice farmlands and lack of good sanitation condition, leptospirosis is an endemic disease. Leptospirosis should be considered in every febrile patient with signs and symptoms of renal and hepatic involvements and disorders. It had proven that delay in diagnosis and treatment in leptospirosis could result to multiple organ failure and death (1, 2). Based on literature, acute renal failure in leptospirosis can lead to death but if the patient survives, it is reversible (1-4). But in our practice, we found several cases with irreversible renal function over one year follow-up. So, in this study, the clinical and laboratory features of renal complication of leptospirosis and their outcomes were evaluated.

## Methods

This descriptive follow-up study was performed on all confirmed cases of leptospirosis and ARF who were admitted in Razi Hospital of Mazandaran University of Medical Sciences during a five- years-period (2007-2012). We evaluated all patients with ARF (defined as a serum creatinine (cr)>1.5mg/dl) and presence of one of the following: 1. fever, 2. hepatic disease, 3. bleeding disorders and thrombocytopenia, with history of animal exposure or work in farmlands. 

All the cases were confirmed by enzyme-linked immunosorbent assay (ELISA) for evaluation of leptospira’s IgM and microscopic agglutination test (MAT). ELISA test was done in the northern branch of Pasteur Institute which uses the non-pathogenic strain of leptospira biflexa. MAT test was done by Razi Vaccine and Serum Research Institute. A positive MAT test was defined as a titer of ≥1: 800 in acute phase serum.

Data were collected for demographic characteristics, and presence or absence of following clinical and laboratory manifestations. Fever and chills (ague), jaundice, ecchymosis and petechiae, mucosal bleeding, myalgia, arthralgia, lymphadenopathy, cough, pharyngitis, blood pressure, nausea and vomiting, diarrhea, abdominal pain, hepatomegaly, splenomegaly, oliguria, or anuria (defined as urinary output of<500 ml and 100 ml.24 h, respectively), headache, meningismus and disturbed consciousness were recorded. For each patient the time between disease onset to correct diagnosis was determined.

**Laboratory tests including **serum urea and creatinine, serum Na, K, bilirubin, ALT and AST, proteinuria (24-h-collection), hematuria, serum amylase and lipase, serum creatine-kinase, blood count and blood film, fibrinogenemia, APTT, ACT and prothrombin time were performed at a mission and three months later. All the patients who had abnormal renal function test at third month were followed-up for one year for their renal function tests.

The outcome of patients were classified as 

1. Full recovery of renal (serum creatinine <1.5mg/dl) and hepatic functions (normal bilirubin and transaminases), 

2. Full recovery of hepatic function with mild persistent renal failure (plasma creatinine>1.5mg/dl)),

3. Persistent hepatic and renal dysfunction, and 

4. Death.

Treatment of patients and laboratory investigation particularly serum creatinine and the status of urine were continued over the follow-up period and homodialysis was also performed as clinically indicated. Statistical analysis was performed using t-test for quantitative and chi-square test and Fisher’s exact test for proportions. SPSS Version 11 was used for analysis

## Results

Fifty one patients with ARF due to severe disease (total of 670 confirmed cases of leptospirosis) were treated in the Infectious Diseases Ward of Razi Teaching Hospital between January 1, 2007 and December 31, 2012 (incidence of renal complication was recorded (7.6%)). Most patients were men (82.4%) and the mean age was 53.5±14.8 (22-79) years (table 1). Most of the patients were admitted during summer (rice cultivation season). The predominant symptom was fever, although the length varied between 1-21 days, followed by nausea and vomiting, muscle pain and jaundice. Clinical presentation of patients with leptospirosis and ARF (percentages showing the prevalence of various clinical features in the study population) was presented in table 1. Leukocytosis (>10.000/ml) was common (47%). Thrombocytopenia (< 150.000/ml) occurred in 28 (55%) patients.

**Table 1:** Patient Characteristic and symptoms (n=51

**characteristic**	**Number (%)**
Nausea/vomiting	35(68.6 )
Muscle pain	33 (64.7)
Jaundice	39 (76.5)
Headache	22 (43.1)
Fever&Chills	43(84.7)
Cough	11 (21.6)
Conjunctival suffusion	27 (52.9)
Rash	8 (15.7)
Altered Mental state	5 (9.8)
Oliguria	16 (31)
Anuria	4 (7)

**Figure 1:**
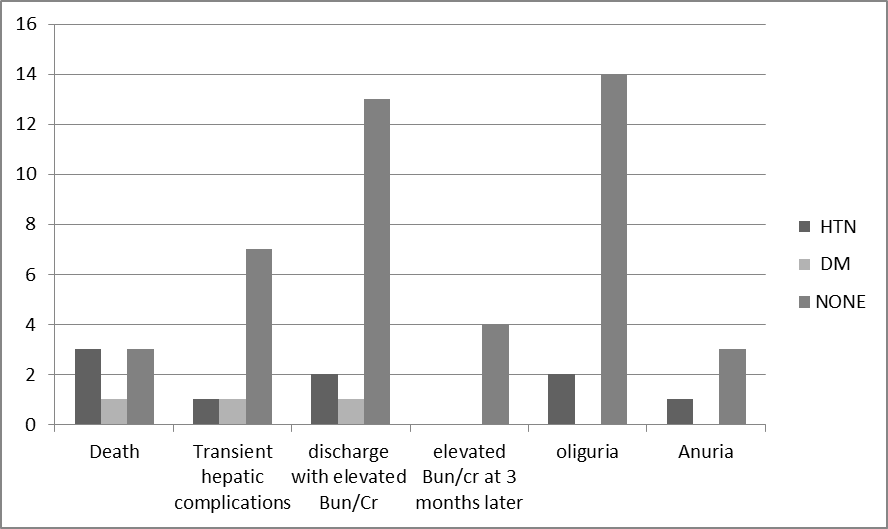
patients underlying disease

Further laboratory tests showed slight increase of hepatic enzymes and bilirubin level (82.4%). Marked elevations were also observed in the blood urea and creatinine levels (table 2). Among the presenting symptoms and laboratory tests, only leukocyte amount showed significant correlation with ARF (table 3). The most frequent underlying diseases were hypertension in 9 and diabetes in 4 patients (figure 1).

**Table 2:** Lab data at admission time, discharge time and 3 months later (7 patients died)

**3 months later** **n=44**	**Discharge** **n=44**	**Admission** **n=51**		
0 44 (100%)0	0 38 (86.4%) 6 (13.6%)	1 (2%) 26 (51%) 24 (47%)	Leucopenia(<4500) Normal(4500-10500) Leukocytosis(>10500)	WBC Count
0	10 (22.7%)	28 (55%)	Thrombocytopenia (<150000)	
0	10 (22.7%)	49 (96%)	(SGOT) AST>35	
0	8 (18.2%)	42 (82.4%)	(SGPT) ALT>35	
0	3 (6.8%)	46 (90.2%)	ALP>147	
0	8 (18.2%)	39 (76.5%)	Total Bil>1.5	
0	8 (18.2%)	36 (70.6%)	Direct Bil0.5	
-	7 (16%)	27 (52.9%)	CPK>400	
0	1 (2.4%)	6 (11.8%)	PT>15	
4 (9.9%)	16 (36.4%)	51 (100%)	BUN>27	
4 (9.9%)	16 (36.4%)	51 (100%)	Cr>1.5	

**Table 3:** Comparison of Key indicators

	**Complete recovery of renal complication (n=41) **	**Death** **(n=7)**	**P value**	**Final outcome (CRF)** **(n=3)**	**P value**
Leukocytosis	34%	100%	P<0.05	100%	P<0.05
Mean age	53.7±16	58.42±13	P>0.05	54.3±3.5	P>0.05
Delay in hospitalization	6.8±5.1	7.6±5.5	P>0.05	7.5±5.9	P>0.05
HTN	14.6%	42.8%	P>0.05	-	P>0.05
DM	7.3%	14.3%	P>0.05	-	P>0.05
oliguria	26.8%	57.1%	P<0.05	33.33%	P>0.05
Anuria	2.4%	28.5%	P<0.05	33.33%	P<0.05

**Renal dysfunction**

All cases presented with ARF symptom. Considerably, only 4 of 51 patients had anuria and 16 cases of them had oliguria. The duration of the anuric phase on average was 6.4 days. Significant hypokalemia (3 mmol) was seen in only 5 (9.8%) patients who were then admitted. Microscopic hematuria was encountered in 25 (49%) and a mild proteinuria (1 g, 2 g/24 h) in 23 (45%) patients. 15 (29.4) patients required dialysis, five of them died and four patients remained with elevated level of urea /creatinine at discharge time (mild persistent renal dysfunction). 

**Treatment and Outcome**

About 90% of patients were treated with ceftriaxone and ampicillin. The other antibiotics used included doxycycline, ciprofloxacin in small percentage. At discharge time, 28 (54.5%) patients were discharged in full recovery and 16 of them remained with mild elevation of liver enzyme and urea/creatinine. There were seven (13.7%) deaths wherein two patients died because of respiratory failure due to pneumonitis, and the others died of renal failure and disseminated intravascular coagulation. After one year follow-up study, two patients remained with creatinine level of more than 1.5.

## Discussion

Clinical manifestations of leptospirosis vary from subclinical infection to severe and potentially fatal disease. 5 to 10 percent of infections could lead to multiple organ damages of kidney, liver and lungs (5). The most severe form is Weil’s syndrome, which is presented by febrile illness with bleeding tendency, hepatic dysfunction and acute renal failure (ARF) (6).

Tubulo-interstitial nephritis is the main cause of acute renal injury in leptospirosis (7, 8). Among 670 confirmed cases of leptospirosis, 51 (7.61%) patients showed a high plasma creatinine level and thus, were diagnosed as ARF. The age range of samples in this study was similar to other studies which were conducted in different years (6, 9).

Male predominance was very common in our patients, actually due to the risk of occupational exposure, especially those working in rice farmlands. Notably, severe leptospirosis usually presents with fever, muscle pain, conjunctival suffusion, jaundice and renal failure (1, 6, 9-12). One of the most common clinical symptoms of this infection in patients was ague (fever and chills) and the second symptom was jaundice at the time of admission (table 1). High total bilirubin level might also be a warning sign, especially oliguric renal failure may result in secondary toxic effects of bilirubin. An important problem regarding the study population is related to delayed diagnosis. Most patients had fever for one week or more before diagnosis, this issue may impose patients at greater risk of acute renal failure. Most patients with acute renal failure have usually significant hepatic involvement (9). Jaundice occurs between the fourth and sixth day but may occur as early as the second day or as late as the ninth day (5). 82.4% of our patients had elevated ALT and total bilirubin above 1.5 was reported in 76.5% of them.

Leptospirosis oliguria primarily occurs after dehydration (1, 10). From this point, renal failure that caused by leptospirosis can be divided into two types of oliguric and non-oliguric which in most cases non-oliguric type can be seen (10). In this case, (non-oliguric) usually creatinine is less than 4 mg/dl (13). Oliguria and anuria in severe cases (with creatinine more than 4) are usually accompanied by dehydration, jaundice and severe sepsis.

Furthermore, coexistent hematuria and rhabdomyolysis is a risk factor for disease progression. Therefore in addition to direct effects of leptospirosis on kidney, jaundice, dehydration and rhabdomyolysis may also affect the course of renal failure (1, 10, 14). Leukocytosis was one of the significant risk factors in predicting of ARF in our leptospirosis cases (table 3). Markum also found this correlation in review of 68 patients with ARF due to leptospirosis (5). It could be the reflection of inflammation grade in patients with acute tubular injury. Daher et al. find no relationship between leukocytosis and acute renal failure (15).

But a few studies showed that leukocytosis is an important factor in predicting the outcome of patients (5, 6). According to Covic's study, there was significant correlation between leukocytosis and deaths (6). In the present, study seven deceased patients had leukocytosis at the time of admission. Among the 16 patients who were discharged with a complication, 13 patients had leukocytosis which persisted until next blood test which was performed three months later. The findings indicate a significant relationship between leukocytosis that may exert a predictive ability for renal outcomes in leptospirosis. Several antibiotics can be used for the treatment of leptospirosis and at present it is difficult to justify about the preferred antibiotic for initiation of treatment (16). There is no relationship between the type of antibiotic and renal outcomes. The result of one study showed superiority peritoneal dialysis to other treatment. In reducing acidosis and faster decline of creatinine levels (17). In our cases, except for two patients, survival rate was similar to other studies on leptospira ARF series (1, 2, 6, 12, 17-23). The results of this study indicate that renal failure may develop in a significant proportion of leptospirosis and recovery of renal function may last several months. Leukocytosis at the time of admission is a risk factor for the development as well as the persistence of renal failure. 

## Notes

**Funding:** This project was financially supported by Mazandaran University of Medical Sciences (Project code: 90179)
